# Remote Sensing Monitoring and Assessment of Global Vegetation Status and Changes during 2016–2020

**DOI:** 10.3390/s23208452

**Published:** 2023-10-13

**Authors:** Li Li, Xiaozhou Xin, Jing Zhao, Aixia Yang, Shanlong Wu, Hailong Zhang, Shanshan Yu

**Affiliations:** State Key Laboratory of Remote Sensing Science, Aerospace Information Research Institute, Chinese Academy of Sciences, Beijing 100101, China; lilifs@aircas.ac.cn (L.L.); zhaojing1@radi.ac.cn (J.Z.); yangax@radi.ac.cn (A.Y.); wsl0579@163.com (S.W.); zhanghl@aircas.ac.cn (H.Z.); yuss@radi.ac.cn (S.Y.)

**Keywords:** vegetation status, global distribution, leaf area index, remote sensing monitoring

## Abstract

Vegetation plays a fundamental role within terrestrial ecosystems, serving as a cornerstone of their functionality. Presently, these crucial ecosystems face a myriad of threats, including deforestation, overgrazing, wildfires, and the impact of climate change. The implementation of remote sensing for monitoring the status and dynamics of vegetation ecosystems has emerged as an indispensable tool for advancing ecological research and effective resource management. This study takes a comprehensive approach by integrating ecosystem monitoring indicators and aligning them with the objectives of SDG15. We conducted a thorough analysis by leveraging global 500 m resolution products for vegetation Leaf Area Index (LAI) and land cover classification spanning the period from 2016 to 2020. This encompassed the calculation of annual average LAI, identification of anomalies, and evaluation of change rates, thereby enabling a comprehensive assessment of the global status and transformations occurring within major vegetation ecosystems. In 2020, a discernible rise in the annual Average LAI of major vegetation ecosystems on a global scale became evident when compared to data from 2016. Notably, the ecosystems demonstrating a slight increase in area constituted the largest proportion (34.23%), while those exhibiting a significant decrease were the least prevalent (6.09%). Within various regions, such as Eastern Europe, Central Africa, and South Asia, substantial increases in both forest ecosystem area and annual Average LAI were observed. Furthermore, Eastern Europe and Central America recorded significant expansions in both grassland ecosystem area and annual average LAI. Similarly, regions experiencing notable growth in both cropland ecosystem areas and annual average LAI encompassed Southern Africa, Northern Europe, and Eastern Africa.

## 1. Introduction

Vegetation is an important component of terrestrial ecosystems, playing a crucial role in climate regulation, the water cycle, soil conservation, and other aspects. It provides essential ecosystem services [1], and also provides habitats for many threatened and endangered species. However, at present, many vegetated areas are threatened by continuous deforestation, overgrazing, fires, climate change, and various other factors, leading to the degradation of vegetation ecosystem services [2]. Therefore, continuous large-scale monitoring of vegetation conditions is needed.

Remote sensing monitoring of vegetation ecosystem conditions and change characteristics has become an essential tool for ecological research and resource management. In September 2015, the United Nations adopted the “2030 Agenda for Sustainable Development” during the Sustainable Development Summit, which set 17 Sustainable Development Goals (SDGs) to be achieved by 2030 [3]. The results of ecological environmental remote sensing monitoring for vegetation ecosystems can provide accurate and effective information support for countries to fulfill their SDGs on sustainable development, ecological environmental protection, and the development and utilization of natural resources.

Remote sensing indicators of vegetation conditions are often used for ecological and biodiversity monitoring. As early as 1968, Carneggie released a report on the application of remote sensing in forestry, discussing methods and technological systems for forestry resource management and monitoring using remote sensing data [4]. Noss proposed a set of long-term biodiversity monitoring indicators, categorized into compositional, structural, and functional indicators, and the methods for obtaining these indicators include ground measurements and remote sensing [5]. Murtaugh subsequently proposed a statistical assessment system for ecological indicators [6]. Lawley reviewed ground measurement and remote sensing methods and indicators used in Australia’s vegetation condition monitoring program, suggesting that a monitoring approach combining ground observations and remote sensing can accurately describe vegetation conditions. Furthermore, the integration of multiple sources and scales, including data from unmanned aerial vehicles and laser scanning, can provide important data support for natural resource management in the future [2]. Tehrany reviewed various methods and attributes for assessing vegetation conditions, including vegetation cover, canopy height, and leaf area. Remote sensing methods can efficiently obtain information on vegetation attributes, but they also have limitations in terms of observation capabilities, and cannot observe under cloudy conditions. However, with the continuous development of remote sensing technology, these issues are gradually being addressed [7]. Oliver proposed a new vegetation integrity index for quantifying the loss and benefits of terrestrial biodiversity value, which can better capture the diversity and complexity of vegetation composition and structure [8].David reviewed the analytical research on dry forests in southern Africa from 1997 to 2020. The combination of remote sensing technology helps assess and monitor forest ecosystems, and the most commonly used products include biomass, LAI, NDVI, etc., in addition to long-term monthly data with high resolution, is the future of forest dynamic monitoring [9].

Vegetation condition monitoring based on remote sensing data has been applied and developed at different regional scales worldwide. Zainal assessed the potential of Landsat satellite data for monitoring changes in marine habitats of coral reefs along the eastern coast of Bahrain [10]. Sims and Colloff used MODIS NDVI data to conduct long-term monitoring of vegetation in floodplain areas of the Paroo River wetlands in Australia, quantifying the vegetation’s response to flooding [11]. Karfs generated land condition monitoring data using Landsat TM and SPOT5 satellite data and conducted comprehensive land monitoring in two pastoral regions of Queensland, Australia [12]. Willis applied multi-source remote sensing data to detect changes in land use, land cover, vegetation phenology, and other key parameters in U.S. protected areas [13]. Pond monitored forest cover changes in Ontario, Canada, between 1990 and 2000, showing an overall increase ranging from 1.7% to 10.9% [14]. Liu studied the spatiotemporal changes and driving factors of vegetation cover in the “Mountain-Oasis-Desert” coupled system and its 11 subsystems in Xinjiang, China, from 1982 to 2013, using GIMMS-NDVI3g and traditional climate data [15]. Campos used ground-based measurements and Landsat 8 satellite remote sensing data to obtain dry forest composition and structural indicators in Argentina’s Iguazú National Park and its surrounding area, demonstrating that remote sensing data can indicate plant species richness and serve as an effective tool for resource management and conservation [16]. Zhao utilized NDVI, land cover, and climate data to conduct trend analysis and regression analysis, studying vegetation changes in the Guanzhong Basin in northwestern China from 2000 to 2020 and their response to climate change and human activities [17]. Krtalic extracted and analyzed forest cover conditions in Zagreb, Croatia, using Sentinel-2 multi-temporal image data, calculating vegetation indices such as NDVI, RVI, and GRVI, and detecting changes in vegetation index classification values [18]. Suir constructed a method that combined ground observation and remote sensing inversion, used hyperspectral images to estimate wetland plant-community quality and vegetation biomass, and developed a wetland vegetation condition indictor system for three Lake Ontario wetland areas [19]. Kayet evaluated the health of vegetation around a mining area using airborne hyperspectral data, simultaneously quantifying the impact of mining on vegetation health [20]. Amputu developed indicators for potential forage biomass and pasture cover types and used drone data to monitor the semi-arid grassland conditions in central Namibia [21]. Remote sensing techniques have evolved from the initial assessment of vegetation classification and composition indicators to the current precise monitoring of various indicators such as coverage, Leaf Area Index (structural indicators) and primary productivity, vegetation health, and phenology (functional indicators). 

Most of the above-mentioned studies focused on the remote sensing monitoring of ecological environments at small regional scales. Since 2012, the Chinese Ministry of Science and Technology has been publishing the “Global Ecological Environment Remote Sensing Monitoring Report” annually for ten consecutive years, which includes reports for the years 2015, 2017, and 2021 [22,23,24]. These reports describe the remote sensing monitoring of global and regional ecological environments. The “Global Ecosystems and Environment Observation Analysis Report Cooperation for the Year 2017” analyzed indicators such as the annual maximum Leaf Area Index and annual maximum vegetation coverage to assess the vegetation ecosystem status and its changes from 2010 to 2015 in ten regions along the “Belt and Road” initiative [25,26]. The “Global Ecosystem and Environment Observation Analysis Research Cooperation for the Year 2021” analyzed indicators such as the maximum vegetation coverage, annual cumulative net primary productivity (NPP), and vegetation growth condition index, to assess the vegetation growth status and its changes from 2015 to 2020 in 19 global regions, covering both terrestrial and aquatic ecosystems [27]. Starting in 2016, the Aerospace Information Research Institute of the Chinese Academy of Sciences has been continuously releasing the “Green Book of Remote Sensing Monitoring: China Sustainable Development Remote Sensing Monitoring Report,” which focuses on the remote sensing monitoring of vegetation conditions in China [28,29,30,31,32].

## 2. Research Area and Data

### 2.1. Research Area

**Figure 1:**
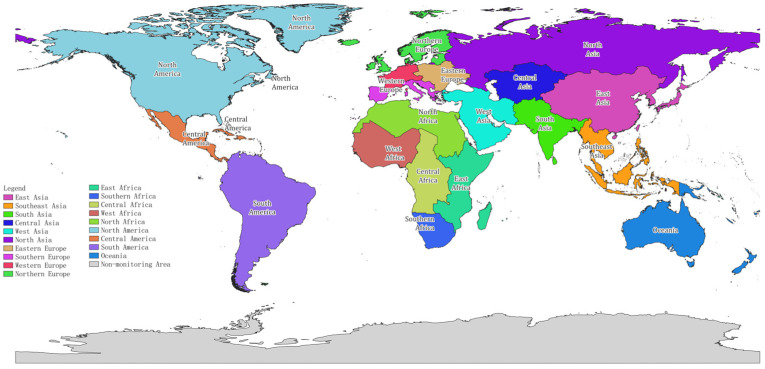
The 19 geographic sub-regions of the world used in the article.

To analyze the status and characteristics of global vegetation ecosystems, this paper divides the global terrestrial ecosystem into 19 geographic regions: East Asia, North Asia, Southeast Asia, South Asia, Central Asia, West Asia, Oceania, Eastern Europe, Northern Europe, Western Europe, Southern Europe, Northern Africa, Eastern Africa, Western Africa, Central Africa, Southern Africa, North America, Central America, and South America. For each of these regions (as shown in Figure 1), indicator calculations and analyses are conducted separately. 

### 2.2. Data

The Leaf Area Index (LAI) is defined as one-half of the total green leaf area per unit horizontal ground surface area [33]. It is an essential parameter for describing the function of vegetation canopies and a significant biophysical parameter that influences vegetation photosynthesis, transpiration, and land surface energy balance. It has been listed as an Essential Climate Variable (ECV) by the global climate change research community [34]. It is not difficult to see from the definition that the LAI is a dimensionless parameter. Based on observational data, the global average LAI values range from 1.98 to 2.31; the annual average LAI based on global remote sensing LAI products is approximately 1.50, and the annual average LAI during the peak growth period reaches approximately 2.0 [35]. LAI is often used in global vegetation change, vegetation phenology investigation, climate change, land surface model, crop yield estimation, biodiversity tracking, forest management, and other fields [35].

This study utilizes the Multi-source data Synergized Quantitative remote sensing production system (MuSyQ) LAI product version 2.0 from 2016 to 2020 for the analysis of the main vegetation ecosystem status and change characteristics. The MuSyQ LAI product was developed by the Aerospace Information Research Institute, Chinese Academy of Sciences. It has a temporal resolution of 5 days and a spatial resolution of 500 m. The product employs an LAI inversion algorithm that considers vegetation mixed pixels and a neural network-based LAI reconstruction algorithm driven by meteorological data. FY-3A/B MERSI data and MODIS data are used synergistically for global LAI inversion and product generation. The MuSyQ LAI product is validated based on high-resolution ground LAI reference maps, and the average relative error is 15.22%, with a validation accuracy of 85% [36,37,38].

In order to extract the main vegetation ecosystems from the global terrestrial ecosystem, this study used the MuSyQ land cover product. The MuSyQ land cover product is constructed using existing 2020 high-resolution global land cover classification products as a sample set. A random forest model is employed, and the product is generated through a combination of global geographical partitioning, sample migration, and model migration methods using FY-3A/B MERSI data and MODIS data. The product includes ten categories: cropland, forest, grassland, shrubland, wetland, water body, tundra, impervious surface, and permanent ice and snow. The overall accuracy of the product’s direct validation in the Chinese region is 90.78%, with a Kappa coefficient of 0.86 [39]. The main analysis subjects chosen in this study are forest ecosystems, grassland ecosystems, and cropland ecosystems. Forest ecosystems are characterized by biotic communities dominated by trees and shrubs, corresponding to the land cover types of forest and shrubland. Grassland ecosystems consist mainly of herbaceous plants and correspond to the land cover types of grassland and tundra. Cropland ecosystems correspond to the land cover type of cropland.

## 3. Method

**Figure 2:**
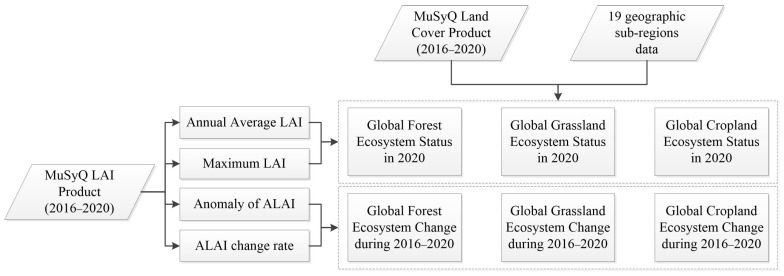
The technical rout of analyze and assessment.

The United Nations’ Sustainable Development Goal (SDG) 15 explicitly focuses on protecting, restoring, and promoting the sustainable use of terrestrial ecosystems, sustainable forest management, combating desertification, halting and reversing land degradation, and halting biodiversity loss. This study combines ecosystem monitoring indicators with the requirements of SDG15. It utilizes a global 500 m resolution vegetation Leaf Area Index product from 2016 to 2020 to calculate annual average Leaf Area Index, anomaly values, and change rates. These indicators are used to assess the status and changes in major vegetation ecosystems globally. The technical route is shown in Figure 2.

(1) Annual Average Leaf Area Index

The annual Average Leaf Area Index (*ALAI*) is used to represent the annual average level of vegetation growth. This study used the MuSyQ LAI global 5-day products to calculate the average Leaf Area Index for the entire globe and 19 regions for the years 2016 and 2020, respectively. The calculation formula is as follows:

(1) $$ALAI=\frac{\sum_{i=1}^{n}LAI_{i}}{n}$$

where *n* represents the number of *LAI* values for a particular pixel in one year, *LAI_i_* refers to the *i*-th value of *LAI*, and *ALAI* is the average *LAI* value for that pixel over the year.

(2) Annual Maximum Leaf Area Index

Using the maximum Leaf Area Index for a year to represent the most vigorous vegetation growth during that year. This article is based on the MuSyQ LAI global 5-day product to calculate the maximum Leaf Area Index for the entire world and 19 regions for the years 2016 and 2020.

(3) Anomaly of the annual average Leaf Area Index

Using the anomaly of the annual average Leaf Area Index (*LAI*) to depict the spatiotemporal variations in vegetation growth. This method refers to the difference between the annual average *LAI* and the long-term average *LAI*. The article is based on the MuSyQ LAI global 5-day product to calculate the annual average *LAI* anomaly for the entire world and 19 regions from 2016 to 2020. The formula for calculating the *LAI* anomaly is as follows:

(2) $$Bias=LAI_{m}-\frac{\sum_{j=1}^{m}LAI_{j}}{m}$$

where *m* represents the number of years, *LAI_j_* denotes the *LAI* value of the corresponding pixel in the *j*-th year, and *Bias* is the anomaly value of that pixel.

(4) Annual average Leaf Area Index change rate

The change rate is used to study the long-term variations in vegetation parameters. Based on the least squares method, the regression line of vegetation’s annual average *LAI* against time is calculated, resulting in a slope image. The specific calculation process involves taking the change rate over the five years for each pixel based on the 2016–2020 MuSyQ LAI product. The formula for calculating the change rate is as follows:

(3) $$K=\frac{m\times \sum_{j=1}^{m}j\times LAI_{j}-(\sum_{j=1}^{m}j)(\sum_{j=1}^{m}LAI_{j})}{m\times \sum_{j=1}^{m}j^{2}-{\left(\sum_{j=1}^{m}j\right)}^{2}}$$

where *m* represents the number of years, which is 5 in this study. *LAI_j_* denotes the vegetation’s Leaf Area Index value of the corresponding pixel in the year, and *K* represents the change trend of that pixel during the five-year period from 2016 to 2020.

To evaluate the annual average Leaf Area Index change rate, this study classifies the change rates into different levels: 0.1<K≤1 indicates a significant increase, 0.01<K≤0.1 indicates a slight increase, −0.01<K≤0.01 indicates no significant change, −0.1<K≤−0.01 indicates a slight decrease, and K≤−0.1 indicates a significant decrease. 

## 4. Monitoring Results

**Figure 3:**
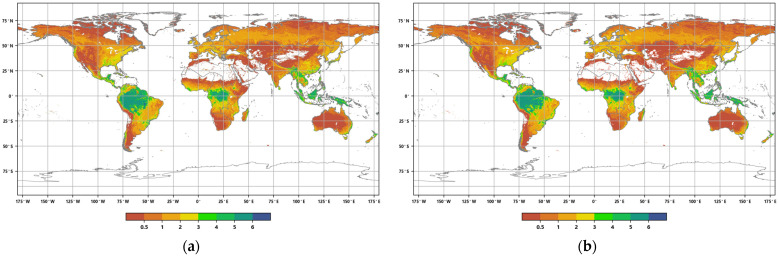
Distribution of global annual ALAI in 2016 (**a**) and 2020 (**b**).

The distribution of the global annual Average Leaf Area Index in 2016 and 2020 (Figure 3) reveals the following: in 2016, the total coverage area of forests, grasslands, and croplands worldwide was 102.929 million square kilometers, accounting for approximately 69.13% of the global land surface area. The maximum value of the annual ALAI was 6.88. By 2020, the global vegetation coverage area decreased to 102.8018 million square kilometers, representing approximately 69.04% of the global surface area. The vegetation coverage area was reduced by approximately 127,200 square kilometers, accounting for approximately 0.85‰ of the global land surface area. The maximum value of the annual ALAI was 6.89. These data indicate that, during this five-year period, global vegetation coverage decreased slightly, while the annual ALAI showed a slight increase.

**Figure 4:**
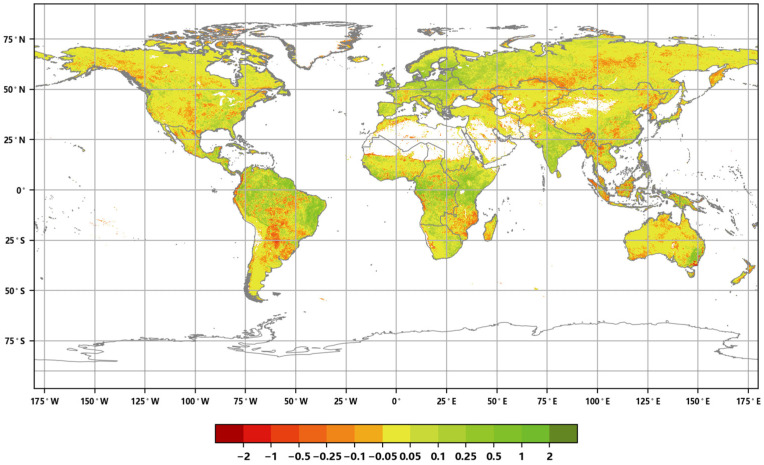
Global annual average LAI anomaly distribution from 2016 to 2020.

**Figure 5:**
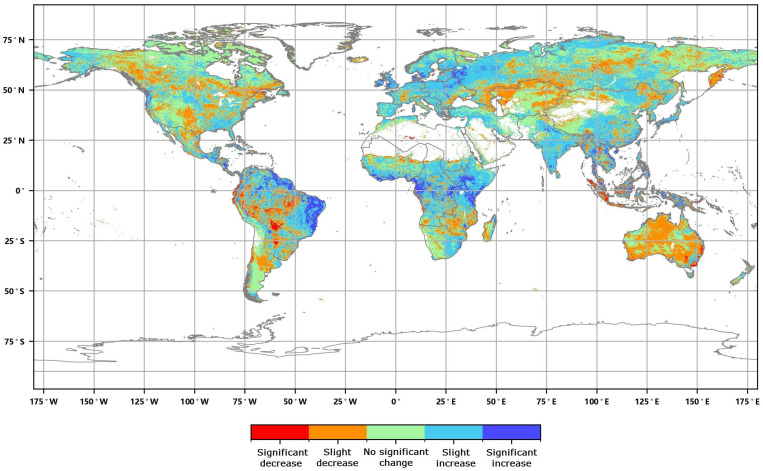
Global annual average Leaf Area Index change rate distribution from 2016 to 2020.

**Figure 6:**
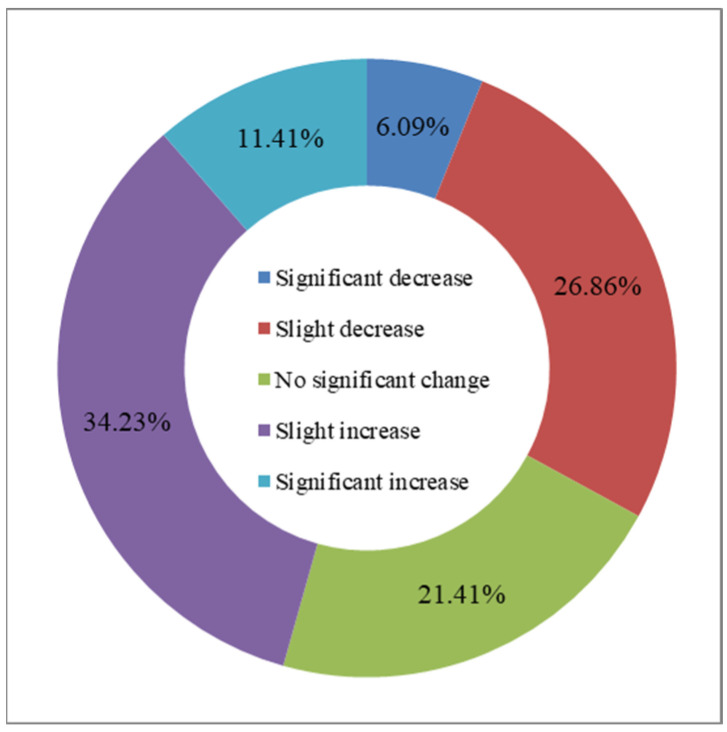
Graded global annual average Leaf Area Index change rate from 2016 to 2020.

The distribution of the global annual average Leaf Area Index anomalies from 2016 to 2020 (Figure 4) shows that the anomalies for the annual average LAI of major vegetation ecosystems ranged from 0.007 to 0.045. The positive anomalies covered approximately 54.86% of the area of major vegetation ecosystems, while the negative anomalies covered approximately 45.14% of the area. Furthermore, the spatial distribution of the global LAI change rates from 2016 to 2020 (Figure 5) indicates that the annual average LAI of major vegetation ecosystems increased during this period, with change rates averaging between 0.2% to 1.9%. The proportion of ecosystems exhibiting slight increases was the highest at 34.23%, while the proportion of significantly decreased areas was the lowest at 6.09% (Figure 6). 

### 4.1. Global Forest Ecosystem Status and Changes

**Figure 7:**
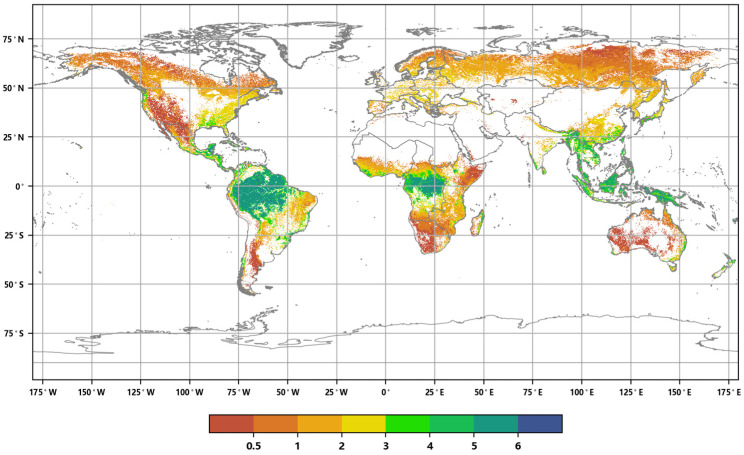
Distribution of annual ALAI Index of the global forest ecosystem in 2020.

**Figure 8:**
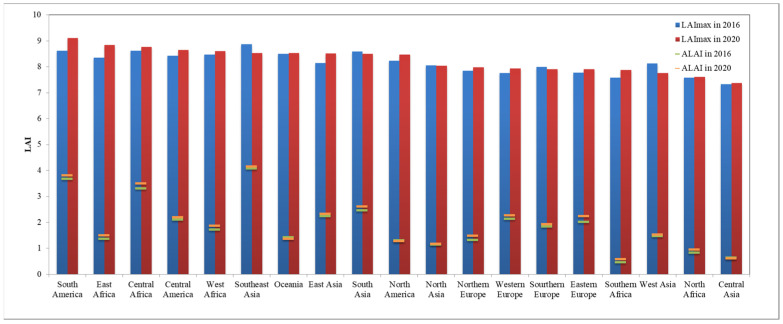
Annual ALAI and maximum LAI of the forest ecosystem in sub-regions for the years 2016 and 2020.

The global forest ecosystem corresponds to forest and shrubland in terms of land cover types and is mainly distributed in Southeast Asia, northern South America, Central Africa, northern Asia, northern North America, and the eastern coastal regions of Oceania. The distribution of the global average Leaf Area Index in 2020 (Figure 7), and the annual average and annual Maximum Leaf Area Index (Figure 8), show extensive tropical rainforests in Southeast Asia, northern South America, and Central Africa, located near the equator. Due to abundant sunlight and water conditions, these tropical rainforests remain evergreen throughout the year, with an annual ALAI Index exceeding 3.5, and the highest value in the Southeast Asia region (4.15). Southern Africa mainly consists of tropical dry forests, where the loss of leaves during the dry season leads to a decrease in the Leaf Area Index. Hence, despite the widespread distribution of tropical dry forests, the annual ALAI is the lowest in Southern Africa (0.57). Boreal forests, apart from tropical rainforests, are the most extensive forest type and are mainly found in northern Europe, northern Asia, and northern North America. These forests consist mainly of evergreen coniferous trees due to their long and cold winters, resulting in a relatively lower Leaf Area Index ranging from 1.17 to 1.48. However, they accumulate a high above-ground biomass due to the long lifespan of tree species.

**Figure 9:**
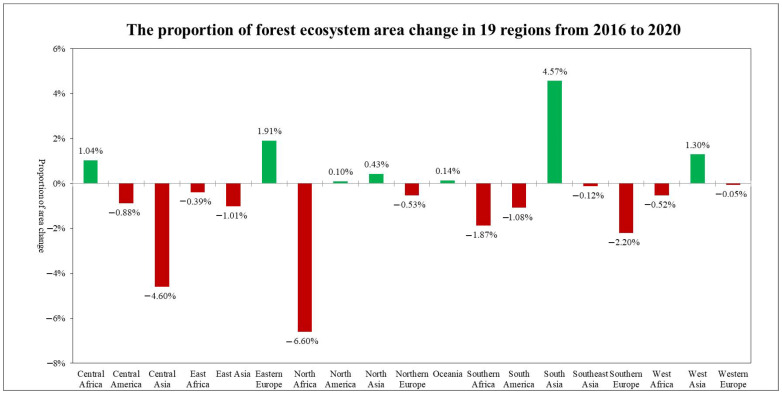
The proportion of forest ecosystem area change in 19 regions from 2016 to 2020.

**Figure 10:**
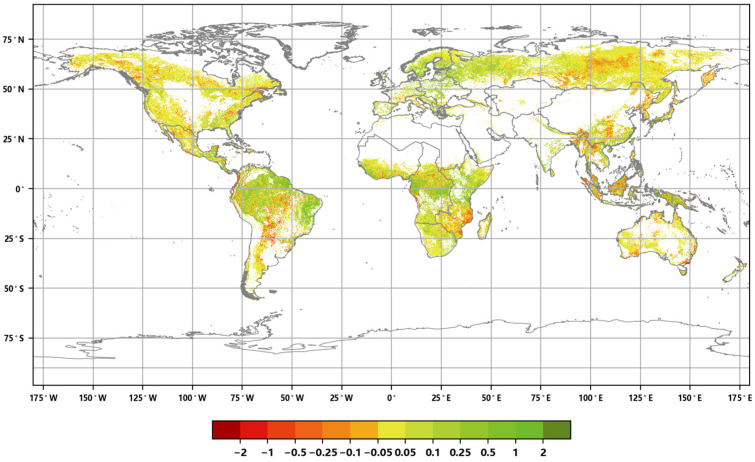
Annual average LAI anomaly distribution of the global forest ecosystem from 2016 to 2020.

**Figure 11:**
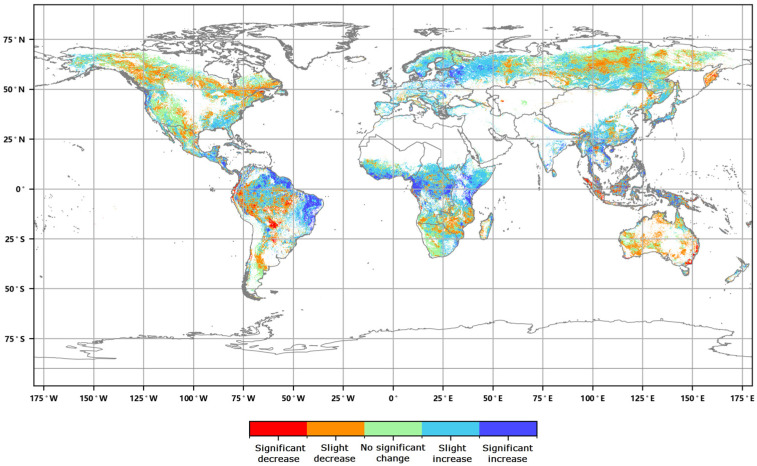
Global annual average Leaf Area Index change rate of the global forest ecosystem from 2016 to 2020.

**Figure 12:**
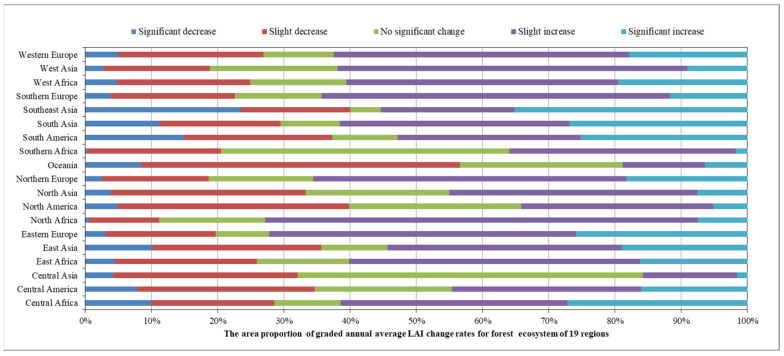
The area proportion of graded annual average LAI change rates for the forest ecosystem of 19 regions.

In 2020, among the 19 global geographical sub-regions, 12 of them experienced a decrease in the area of forest ecosystems compared to 2016. The region with the highest proportion of forest area reduction was Northern Africa (6.6%), followed by Central Asia (4.60%). Conversely, the regions with the highest proportion of forest area increase were South Asia (4.57%) followed by Eastern Europe (1.91%) (Figure 9).The spatial distribution maps of the global annual average Leaf Area Index anomalies and change rates for the period 2016–2020 (Figure 10 and Figure 11 and Table 1) show that the regions with a significant increase in forest LAI included Eastern Europe (with a change rate of 5.3%), Central Africa (4.98%), and Western Africa (4.55%), among others. Regions with a significant decrease in forest LAI included Oceania (−1.77%), Central Asia (−0.84%), and North America (−0.11%), among others. The regions with the highest proportion of significant forest area increase were Southeast Asia (35.15%), Central Africa (27.22%), and South Asia (26.88%), while the regions with the highest proportion of significant forest area decrease were Southeast Asia (23.48%), South America (14.95%), and South Asia (11.16%) (Figure 12).

Eastern Europe, Central Africa, and South Asia showed significant increases in both the forest ecosystem area and annual ALAI. This can be attributed to the proactive reforestation and afforestation policies adopted by various countries in these regions, as well as forest protection efforts like land restoration. Additionally, the rising temperatures and increased precipitation in recent years has provided favorable conditions for forest growth in Eastern Europe. On the other hand, both Central Africa and Central Asia experienced noticeable decreases in both forest ecosystem area and ALAI.

### 4.2. Global Grassland Ecosystem Status and Changes

**Figure 13:**
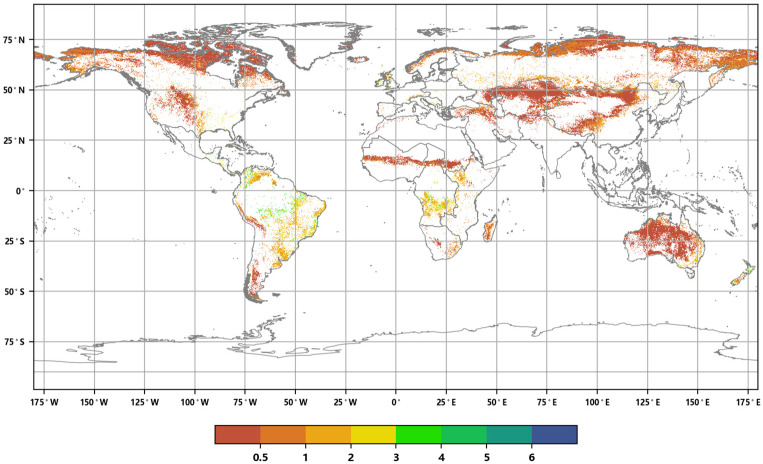
Distribution of annual ALAI of the global grassland ecosystem in 2020.

**Figure 14:**
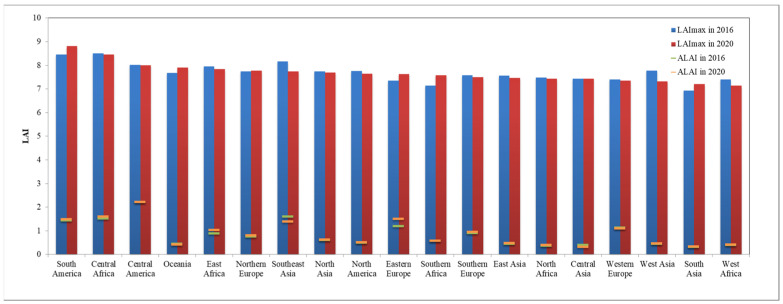
Annual average LAI and maximum LAI of the grassland ecosystem in different regions for the Years 2016 and 2020.

The grassland ecosystem, corresponding to the surface cover types of grasslands and tundra, is mainly distributed in regions such as Northern Asia, Oceania, South America, North America, East Asia, Central Asia, and Central Africa. The spatial distribution of the global average Leaf Area Index for grasslands in 2020 (Figure 13) and annual average and annual maximum LAI (Figure 14) show that grassland ecosystems have lower LAI values compared to forest ecosystems, with annual averages ranging from 0.32 to 2.22. The region of Central America exhibits the highest annual ALAI value for grassland ecosystems (2.22), followed by Central Africa (1.60). Regions with higher LAI values are primarily located at the border zones between tropical rainforests and tropical grasslands, where precipitation is abundant, and the grasslands are dense. Central Asia has the lowest annual ALAI value (0.32), followed by South Asia (0.33). Regions with lower LAI values are mainly situated closer to deserts, characterized by limited precipitation and arid climates, resulting in sparse and short grasslands.

**Figure 15:**
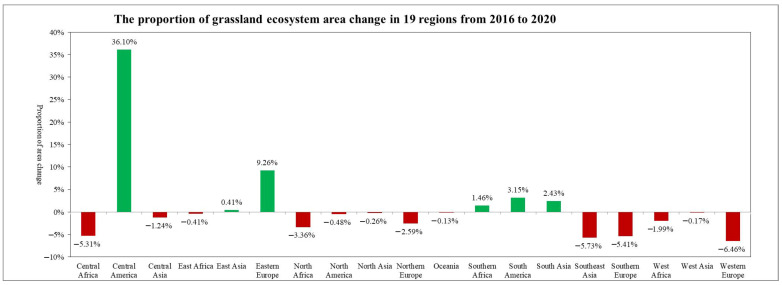
The proportion of grassland ecosystem area change in 19 regions from 2016 to 2020.

**Figure 16:**
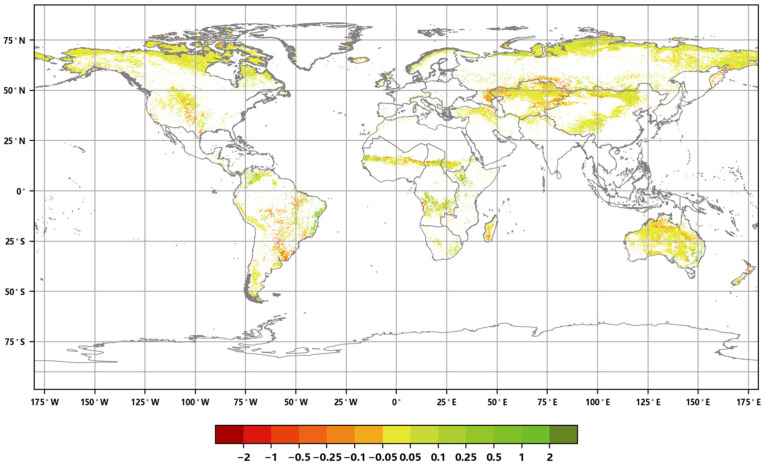
Annual average LAI anomaly distribution of the global grassland ecosystem from 2016 to 2020.

**Figure 17:**
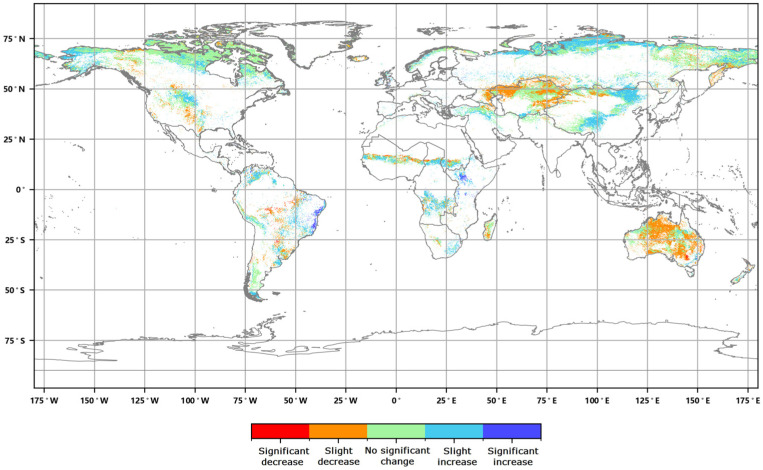
Global Annual average Leaf Area Index change rate of the global grassland ecosystem from 2016 to 2020.

**Figure 18:**
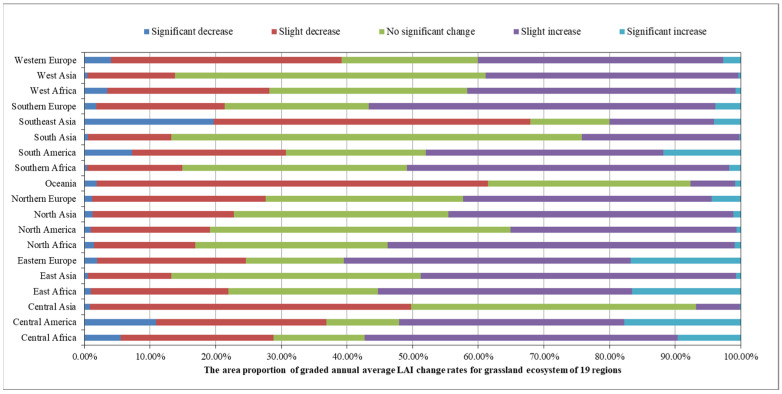
The area proportion of graded annual average LAI change rates for the grassland ecosystem of 19 regions.

In 2020, among the 19 global geographical sub-regions, 13 experienced a decrease in the area of grassland ecosystems compared to 2016. The region with the highest proportion of grassland area reduction was Western Europe (6.46%), followed by Southeast Asia (5.73%). On the other hand, the regions with the highest proportion of grassland area increase were Central America (36.10%) followed by Eastern Europe (9.26%) (Figure 15). The spatial distribution maps of the global annual average Leaf Area Index anomalies and change rates for grassland ecosystems during the period 2016–2020 (Figure 16 and Figure 17 and Table 2) show that regions with a significant increase in grassland LAI included Eastern Europe (with a change rate of 3.61%), Eastern Africa (3.56%), and Central Africa (1.78%), among others. Regions with a significant decrease in grassland LAI included Southeast Asia (−5.08%), Oceania (−1.75%), and Central Asia (−1.47%), among others. The regions with the highest proportion of significant grassland area increase were Central America (17.76%), Eastern Europe (16.77%), and Eastern Africa (16.54%), while the regions with the highest proportion of significant grassland area decrease were Southeast Asia (19.70%), Central America (10.93%), and South America (7.28%) (Figure 18).

Eastern Europe and Central America showed significant increases in both the grassland ecosystem area and annual ALAI, while Southeast Asia, Central Asia, and Western Europe showed noticeable decreases in both grassland ecosystem area and annual ALAI. These trends are closely related to the changes in global precipitation. Currently, regions such as Oceania and Central Asia are experiencing a decrease in precipitation, leading to increased aridity and a subsequent reduction in grassland LAI. Conversely, higher latitudes like Eastern Europe are experiencing temperature increases and higher precipitation, leading to an increase in grassland LAI.

### 4.3. Global Cropland Ecosystem Status and Changes

**Figure 19:**
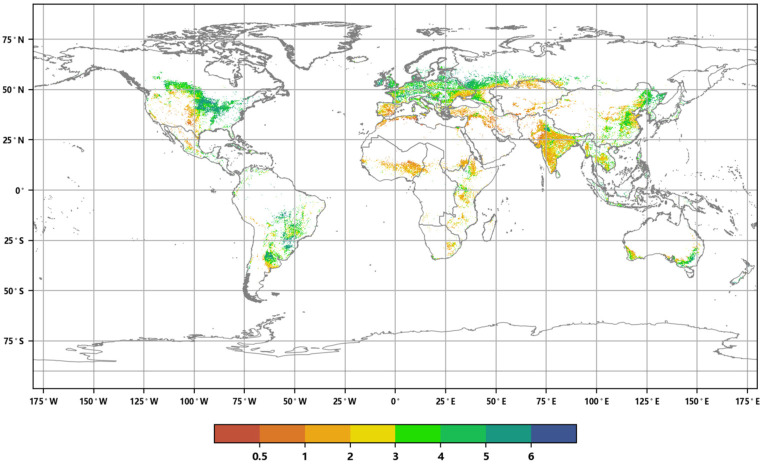
Distribution of annual maximum Leaf Area Index of the global cropland ecosystem in 2020.

The global cropland ecosystem is mainly distributed in regions such as South Asia, East Asia, South America, Northern Asia, and Eastern Europe. The spatial distribution of the global annual Maximum Leaf Area Index for cropland in 2020 (Figure 19) and the table of annual maximum LAI values (Table 3) show that the cropland ecosystem’s LAI is generally lower than that of forest and grassland ecosystems due to the seasonal cultivation of crops. The average LAI values range from 0.43 to 1.81. The maximum LAI values range from 7.17 to 8.57. The region of Northern Europe exhibits the highest annual ALAI value for cropland (1.81), followed by Southeast Asia (1.54). The region of South America exhibits the highest annual maximum LAI value for cropland (8.57), followed by Oceania (8.24). Conversely, the regions of Central Asia and Western Asia have the lowest annual ALAI values (0.43 and 0.50, respectively). The lowest annual maximum LAI values are in Central Asia and Southern Europe (7.17 and 7.40 respectively). Regions with higher LAI values are typically characterized by warm and humid climates, abundant rainfall, and intensive crop cultivation. In contrast, regions with lower LAI values are mostly found in arid areas with high temperatures and limited precipitation, which are unfavorable for crop growth.

**Figure 20:**
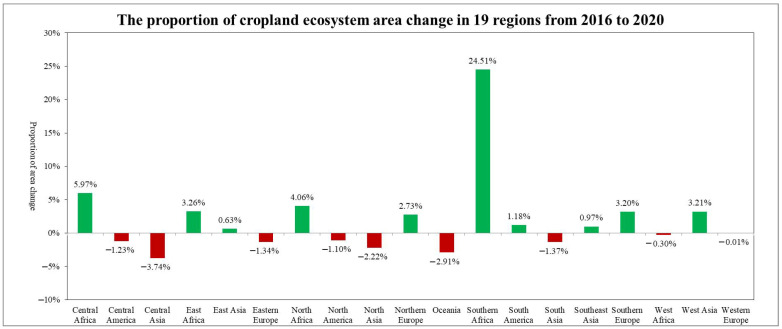
The proportion of cropland ecosystem area change in 19 regions from 2016 to 2020.

**Figure 21:**
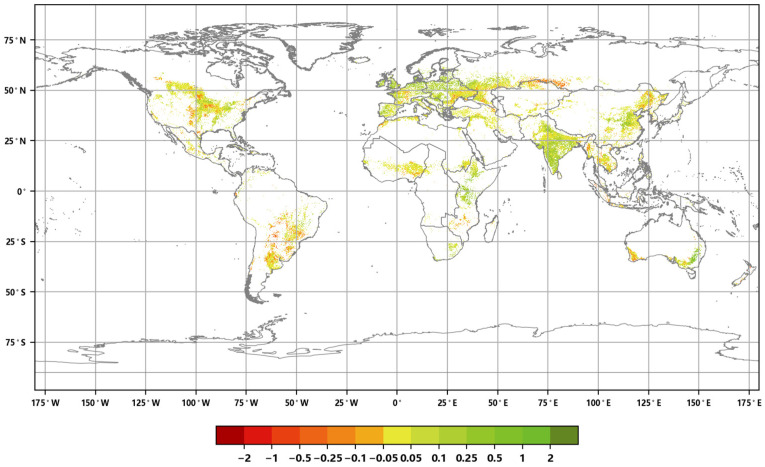
Annual average LAI anomaly distribution of the global cropland ecosystem from 2016 to 2020.

**Figure 22:**
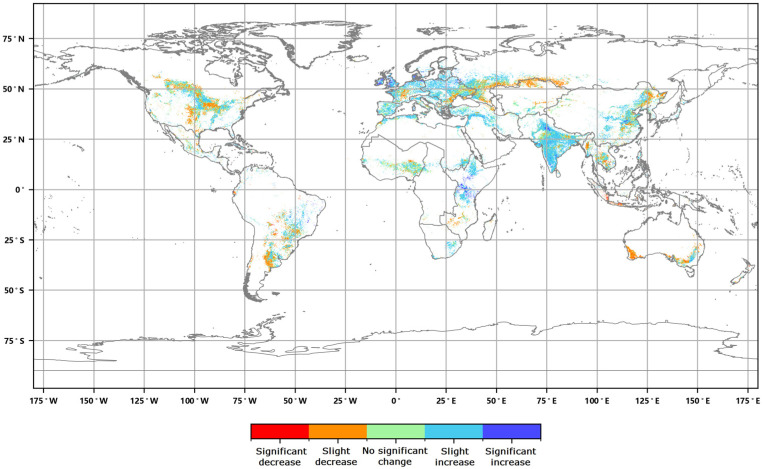
Global annual average Leaf Area Index change rate of the global cropland ecosystem from 2016 to 2020.

**Figure 23:**
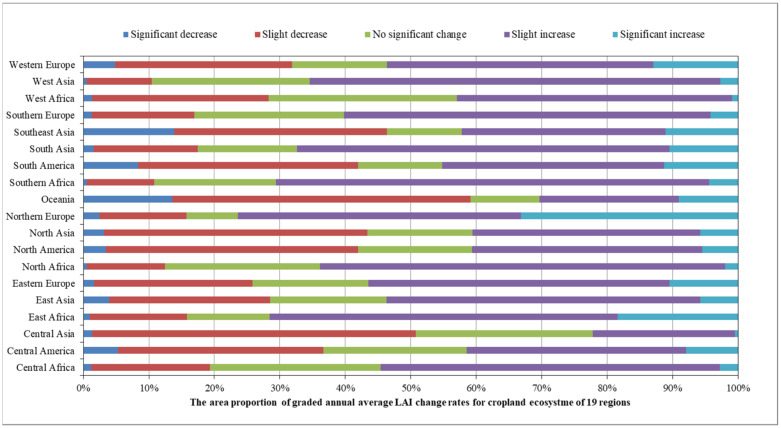
The area proportion of graded annual average LAI change rates for the cropland ecosystem of 19 regions.

In 2020, 9 of the 19 global geographical sub-regions experienced a decrease in the area of cropland ecosystems compared to 2016. The region with the highest proportion of cropland area reduction was Central Asia (3.74%), followed by Oceania (2.91%). The regions with the highest proportion of cropland area increase were Southern Africa (24.51%), followed by Central Africa (5.97%) (Figure 20). The spatial distribution maps of the global annual average Leaf Area Index anomalies and change rates for cropland ecosystems during the period 2016–2020 (Figure 21 and Figure 22 and Table 4) show that regions with a significant increase in cropland LAI included Northern Europe (with a change rate of 7.60%), Eastern Africa (4.69%), and South Asia (3.30%), among others. Regions with a significant decrease in cropland LAI included Oceania (−1.86%), Central Asia (−1.29%), and Southeast Asia (−1.08%), among others. The regions with the highest proportion of significant cropland area increase were Northern Europe (33.15%), Eastern Africa (18.34%), and Western Europe (12.91%), while the regions with the highest proportion of significant cropland area decrease were Southeast Asia (13.91%), Oceania (13.59%), and South America (8.34%) (Figure 23).

Cropland ecosystems in Southern Africa, Northern Europe, and Eastern Africa showed significant increases in both area and annual ALAI, while Oceania and Central Asia experienced noticeable decreases in both cropland area and annual ALAI. The increase in cropland area in Northern Europe, mainly concentrated in countries like Denmark and Sweden, is attributed to the government’s strong focus on agriculture and the implementation of policies like the “Agriculture Law” to protect agricultural land. Conversely, regions in Western Oceania and Central Asia have experienced severe drought due to continuous declines in precipitation, resulting in a significant reduction in the annual ALAI.

## 5. Conclusions

This paper used MuSyQ global Leaf Area Index products from 2016 and 2020 to calculate the annual Average Leaf Area Index, annual maximum Leaf Area Index, annual average Leaf Area Index anomaly, and annual average Leaf Area Index change rate, and analyzed the global status and changing characteristics of forest, grassland and farmland ecosystems in 19 geographical sub-regions. The results show that in 2020, the annual Average Leaf Area Index of major vegetation ecosystems in the world showed an overall increase compared with 2016. The annual Average Leaf Area Index of global forests, grasslands and cropland ecosystems in 2020 increased compared with 2016, and the increase has reached 5%. The area of forest and grassland ecosystems has decreased compared with 2016. This is due to man-made and natural causes such as deforestation and fires. The monitoring indicators and technical routes used in this article to analyze and monitor the status and change characteristics of the vegetation ecosystem can provide analytical ideas for remote sensing monitoring of the ecological environment at a large regional scale and even globally; the analytical data and conclusions can serve as a basis for judging the quality of the ecological environment and the effectiveness of ecological environment protection. In the future, while continuing to monitor the status and changing characteristics of vegetation ecosystems, we should also add functional indicators and increase the length of time series, and analyze the changing characteristics and causes of various vegetation ecosystems, including global change, climate warming, and other scales.

## Figures and Tables

**Table 1:** List of annual average Leaf Area Index anomalies and change rates of forest ecosystem.

Region	Change Rate	LAI Anomaly	Region	Change Rate	LAI Anomaly	Region	Change Rate	LAI Anomaly
Eastern Europe	5.30%	0.140	East Africa	3.17%	0.060	North Asia	0.98%	0.010
Central Africa	4.98%	0.131	Southeast Asia	3.00%	0.007	Southern Africa	0.80%	0.041
West Africa	4.55%	0.071	Southern Europe	2.77%	0.066	North America	−0.11%	−0.001
Northern Europe	4.13%	0.128	South America	2.61%	0.102	Central Asia	−0.84%	−0.045
South Asia	4.09%	0.086	East Asia	2.52%	0.010	Oceania	−1.77%	−0.033
North Africa	3.62%	0.038	West Asia	2.37%	0.021			
Western Europe	3.28%	0.069	Central America	2.00%	0.040			

**Table 2:** List of annual average Leaf Area Index anomalies and change rates of grassland ecosystem.

Region	Change Rate	LAI Anomaly	Region	Change Rate	LAI Anomaly	Region	Change Rate	LAI Anomaly
Eastern Europe	3.61%	0.119	Northern Europe	1.18%	0.030	West Africa	0.16%	0.022
East Africa	3.56%	0.072	East Asia	1.14%	0.009	Western Europe	−0.22%	0.013
Central Africa	1.78%	0.061	South America	1.06%	0.008	Central Asia	−1.47%	−0.032
Central America	1.61%	0.067	West Asia	0.57%	0.004	Oceania	−1.75%	−0.008
Southern Europe	1.52%	0.057	North Asia	0.52%	0.011	Southeast Asia	−5.08%	−0.083
Southern Africa	1.49%	0.049	North America	0.40%	0.002			
North Africa	1.18%	0.027	South Asia	0.27%	0.000			

**Table 3:** Annual maximum Leaf Area Index and coverage area of the cropland ecosystem in different regions for the years 2016 and 2020.

Region	2016			2020		
	*ALAI*	*LAI*_max_	Area (10^4^ km^2^)	*ALAI*	*LAI*_max_	Area (10^4^ km^2^)
South America	1.36	8.28	176.04	1.38	8.57	178.11
Oceania	1.2	7.99	63.03	1.11	8.24	61.19
East Africa	1.06	8.09	86.21	1.15	8.24	89.02
South Asia	0.79	7.9	259.54	0.93	8.22	255.99
Southeast Asia	1.52	8.3	88.8	1.54	8.21	89.66
Central Africa	0.88	7.78	11.71	0.85	8.03	12.41
Central America	1.37	8.2	33.36	1.28	7.89	32.95
Western Europe	1.37	7.58	58.97	1.49	7.88	58.96
Eastern Europe	0.9	7.64	105.78	0.99	7.82	104.36
Northern Europe	1.47	8.03	32.44	1.81	7.81	33.33
North America	1.02	8.04	242.95	1.02	7.78	240.28
North Asia	0.79	7.75	123.24	0.82	7.78	120.5
East Asia	0.96	7.85	187.3	1.03	7.7	188.48
Southern Africa	0.7	7.13	12.35	0.81	7.59	15.37
West Africa	0.73	7.54	79.51	0.72	7.58	79.27
North Africa	0.47	7.31	46.04	0.55	7.58	47.91
West Asia	0.47	7.52	68.55	0.5	7.57	70.75
Southern Europe	0.9	7.67	53.27	0.95	7.4	54.98
Central Asia	0.5	7.2	39.84	0.43	7.17	38.35

**Table 4:** List of annual average Leaf Area Index anomalies and change rates of the cropland ecosystem.

Region	Change Rate	LAI Anomaly	Region	Change Rate	LAI Anomaly	Region	Change Rate	LAI Anomaly
Northern Europe	7.60%	0.193	North Africa	1.97%	0.017	North Asia	0.28%	0.018
East Africa	4.69%	0.129	Southern Europe	1.95%	0.076	North America	0.25%	0.022
South Asia	3.30%	0.096	Central Africa	1.49%	0.031	Southeast Asia	−1.08%	−0.021
Southern Africa	2.73%	0.094	East Asia	1.27%	0.036	Central Asia	−1.29%	−0.028
Eastern Europe	2.50%	0.077	Central America	0.59%	0.020	Oceania	−1.86%	0.106
Western Europe	2.09%	0.082	West Africa	0.41%	0.000			
West Asia	2.05%	0.026	South America	0.39%	−0.025			

## Data Availability

Not applicable.
